# Characteristics and economic burden of patients with somatoform disorders in Chinese general hospitals: a multicenter cross-sectional study

**DOI:** 10.1186/s12991-023-00457-y

**Published:** 2023-08-12

**Authors:** Xiangyun Yang, Jia Luo, Pengchong Wang, Yue He, Cong Wang, Lijuan Yang, Jing Sun, Zhanjiang Li

**Affiliations:** 1 The National Clinical Research Center for Mental Disorders & Beijing Key Laboratory of Mental Disorders, Beijing Anding Hospital, Capital Medical University, No. 5 Ankang Hutong Deshengmen Wai, Xicheng District, Beijing, 100088 China; 2 Advanced Innovation Center for Human Brain Protection, Capital Medical University, Beijing, China; 3 School of Medicine and Dentistry, Griffith University, Gold Coast, QLD Australia; 4 Institute of Integrated Intelligence and Systems, Griffith University, Gold Coast, QLD Australia

**Keywords:** Somatoform disorders, Self-screening Questionnaire for Somatic Symptoms, Characteristic, Economic burden, General hospital

## Abstract

**Background:**

In China, patients with somatoform disorders (SFD) often seek medical treatment repeatedly in outpatient clinics of general hospitals, which increases unreasonable medical expenses. It is imperative to provide early screening to these patients and specialized treatment to reduce the unnecessary cost. This study aimed to screen patients with SFD in general hospitals using a new Chinese questionnaire and explore the characteristics and economic burden of these patients.

**Methods:**

Patients (n = 1497) from the outpatient department of neurology, cardiology and gastroenterology of three large general hospitals were included. Participants were screened using a newly developed questionnaire, the Self-screening Questionnaire for Somatic Symptoms (SQSS), to identify the patients with SFD (total SQSS score ≥ 29 points). We compared the demographics and clinical information of patients with and without SFD. Logistic regression was used to explore potential factors related to medical expenses, visits to doctors and sick leave days taken.

**Results:**

The frequency of detection of patients with SFD was 17.03%. There were significant differences in employment, doctor visits, symptom duration, medical expenses, sick leave days, PHQ-15 scores, and PHQ-9 scores between patients with SFD and without SFD. General nonspecific somatic symptoms were frequently present in patients with SFD. Several potential factors were associated with higher medical expenses, repeated doctor visits, and sick leave days taken in the regression analysis.

**Conclusion:**

The findings indicate that patients with SFD are common in general hospitals, and their direct and indirect economic burden is higher than that of non-SFD patients, which indicates that more screening effort should be made to this group to early identify their problems. Certain characteristics were identified among patients with SFD and several factors were associated with negative consequences of SFD, all of which might be prevented by developing a preventive intervention program to reduce the economic burden of the patients.

**Supplementary Information:**

The online version contains supplementary material available at 10.1186/s12991-023-00457-y.

## Background

Individuals with somatoform disorder (SFD) experience medically unexplained symptoms (MUS) from various organ’s systems [1, 2]. In many countries, patients with MUS and SFD first seek medical help in the primary care setting [3, 4]. It has been reported that 40–49% of patients from primary care who were screened with questionnaires were considered to have at least one MUS [4]. It has been reported that the prevalence of SFD was 16.1% (95% CI 12.8–19.4) in general practice [3]. However, in China, as a result of the lack of individualised medical systems, these patients mostly visit the outpatient clinics of large public hospitals. Over the past decade, studies of patients with SFD have remained limited and SFD has been severely underdiagnosed in China. A multicentre study investigated the prevalence of SFD in the outpatient departments of 23 general hospitals in the Shenyang province of China, found that the one-month prevalence of any type of SFD was 1.56% [5]. In fact, many patients visiting doctors at general hospitals are at high risk of SFD [6]. However, few patients actively seek, or are referred to, psychiatric/psychological assessment and intervention [7].

Patients with SFD seek health care nearly twice as often as patients with other mental disorders, resulting in increased economic burden and impaired social functioning [8]. In addition to their medical expenses, patients with SFD also encounter indirect costs, such as loss of productivity due to the number of sick leave days they take [9]. It has been reported that the severity of somatic symptoms was positively associated with the direct and indirect costs for patients with SFD [10]. An Indian study showed that patients with SFD experience considerable levels of disability, and that their caregivers go through various levels of burden in daily life; this burden is comparable to the one for schizophrenia [11]. Thus, early screening for patients at high risk for SFD is imperative to improve the diagnosis of SFD and to reduce the associated economic burden. Considering the restrictions on doctors’ consultation time in general hospitals, self-report questionnaires is a convenient tool to assist doctors in detecting symptoms of SFD during the clinics. The Patient Health Questionnaire-15 (PHQ-15) is a measure of somatic symptom severity that includes 15 somatic symptoms, and has been recommended as a screening instrument for SFD [12]. When it is used to identify SFD, a cut-off of 10 points on the PHQ-15 was previously considered as optimal, as this score results in a sensitivity of 80.2% and specificity of 58.5% [13]. However, the specificity of this tool for diagnosing SFD is not ideal, as the PHQ-15 cannot distinguish between medically explained and unexplained symptoms.

Somatisation is a complex concept. In addition to physical symptoms, it also has cognitive and behavioural dimensions [14]. The diagnosis of SFD has been replaced by that of ‘somatic symptom disorder’ (SSD) in DSM-5. SSD involves one or more physical symptoms, accompanied by a significant amount of time, energy, emotions, and/or behaviour associated with the symptoms, leading to severe pain and/or dysfunction [15]. The new diagnostic criteria emphasise the importance of both somatic symptoms and impaired thoughts, emotions and behaviour [16]. However, DSM-IV does not require certain psychological and behavioural characteristics to be present when diagnosing SFD. Therefore, in the future, the questionnaire for screening SSD patient needs to comprehensively reflect the different symptom dimensions of SSD specified in DSM-5. The PHQ-15 only has a single dimension of somatic symptoms and is not able assess psycho-behavioural symptoms. The Whiteley index is another commonly used questionnaire for SFD to assess patients’ disease concerns and beliefs, but this index is not able to assess physical symptoms [17]. Recently, a new Somatic Symptom Scale-China.

(SSS-CN) questionnaire was developed and it consisted of four dimensions: physical disorders, depressive disorders, anxiety disorders, and depression and anxiety disorders based on DSM- 5 [18]. Using the SSS-CN, the research team explored the prevalence of SSD among elder population in large communities, and indicated the prevalence of SSD in the elderly was higher than that in the non-elderly (63.2% vs. 45.3%) [19]. The findings indicate that SSD is a common psychological problem among the elderly population in the community. However, it is worth noting that the purpose of SSS-CN was to screen for general psychological disorders in general hospitals, rather than specifically assessing SSD [18]. Therefore, the high prevalence of SSD found in this study may be related to the insufficient specificity of SSS-CN. Thus, it is necessary to develop a new multidimensional self-report questionnaire to adapt to the evolution of the diagnosis of SFD to SSD.

Based on the clinical and research status of SFD in China, our research team has developed a new multidimensional questionnaire, the self-screening questionnaire for somatic symptoms (SQSS), which aimed to early identify SFD in general hospitals. Although the development of SQSS was based on the diagnostic criteria of DSM- IV for SFD, we also considered the situation where DSM-5 changed SFD to SSD, and included four symptom dimensions of SSD in the scale, namely somatic symptoms, negative perceptions, illness behaviour, and social function. SQSS has good validity and reliability for the patients in general hospitals. The correlation analysis was performed between the SQSS score and the PHQ-15 score, which show a positive correlation (r = 0.683, P < 0.01). The cutoff score of 29 applies to patients with SFD in Chinese general hospitals [20]. Using the SQSS, our current study undertook a multicentre cross-sectional investigation to identify patients with SFD in three large general hospitals in Beijing. The first goal was to determine the detection rate of patients with SFD and explore the characteristics of these patients. In addition to the physical symptoms, patients with SFD often suffer from depressive symptoms. Van den Bergh and colleagues [11] suggested that persons with (MUS) SSD and persons with depression disorder share the trait of dispositional negativity as a general vulnerability for psychopathology. Therefore, this study also used two classic scales, PHQ-15 and PHQ-9, to evaluate the physical symptoms and depressive emotions of the subjects, in order to comprehensively reflect their clinical symptoms. The second goal was to explore whether patients with SFD have higher medical expenses, and whether they use more medical care and take more sick leave days from work, as well as to further examine factors which may be potentially related to these adverse outcomes.

## Methods

### Study design and recruitment

The current multicentre cross-sectional study employed a purposive sampling method. Residents of Beijing who visited three large general hospitals (more than 2,000 beds per hospital) were selected as representative of the population of Beijing. Previous studies have indicated that patients with SFD often visit internal medicine specialties such as neurology, cardiology, and gastroenterology medicine [4]. Given the limitations of our human and material resources, three outpatient departments—neurology, cardiology, and gastroenterology—for this study were selected. From June to September 2017, patients who met the inclusion criteria from aforementioned departments in the three hospitals were continuously recruited for the study. The duration of screening for each department in each hospital was seven consecutive working days. For convenience, the screening order for each hospital and department was decided by the chief investigator and the head of each participating hospital according to their daily work schedules. The head of each hospital coordinated with the leaders of the different outpatient departments and arranged screening sites.

The inclusion criteria for participants were the following (1) outpatients who came to see a doctor in the neurology, cardiology or gastroenterology medicine because of illness; (2) aged 18–65 years; (3) males and females; (4) education levels up to junior high school or above (≥ 9 years for education); (5) permanent residents of Beijing; (6) Patients with independent informed consent ability who can clearly understand questionnaire questions and provide answers. The exclusion criteria were as follows: (1) people who sought treatment for family members; (2) those with communication difficulties, language barriers or writing disorders; (3) people with cognitive impairment, organic brain disorders or dementia; (4) people with a history of serious mental disorders; (5) people with a serious illness who could not complete the questionnaire survey. The assessment instruments used in this study are described below.

### Assessment instruments

#### General information questionnaire

The General Information Questionnaire is a self-compiled questionnaire designed to collect demographic data and consultation information from patients. The demographic data recorded includes gender, age, marital status, employment status, and education level. The information collected with regard to consultation includes the hospital and department visited, whether the visit is a first or repeat consultation, duration of symptoms, medical expenses, and whether sick leave days were requested. Employment status includes employees and non-employees (unemployed and/or retirees). The medical expenses include the examination expenses, medication expenses, hospitalization expenses, and other medical expenses related to the disorder (before medical insurance reimbursement), which are calculated monthly on average.

#### Self-screening questionnaire for somatic symptoms (SQSS)

The questionnaire consists of 22 items and involves four dimensions of measurement, including somatic symptoms, negative perceptions, illness behaviour, and social function. Each item scores from zero to five points, with a total score of 88 points. The full list of items is given in Additional file 1: Table S1. The SQSS was used to assess the somatisation symptoms of participants over the previous four weeks. According to the demarcation score of the SQSS determined in our previous study [20], patients with scores equal to or greater than 29 were considered to meet SFD diagnosis**.**

#### Patient Health Questionnaire-15 (PHQ-15)

The PHQ-15 is a classic self-assessment questionnaire covering 15 physical symptoms, and was widely used to assess the physical symptoms participants experienced over the previous four weeks. Each item scores zero to two points, with a total score of zero to 30 points. According to the total score of the scale, the severity is as follows: no physical symptoms (< 5), possible symptoms [5–9], mild to moderate symptoms [10–14] and severe symptoms (≥ 15). The Chinese version has been proven to be reliable and valid in the Chinese population [21]. This study applied the Chinese version of the PHQ-15 to assess the physical symptoms of all participants and the distribution of somatic symptoms of PHQ-15 in patients with SFD.

#### Patient Health Questionnaire-9 (PHQ-9)

SFD patients often comorbid with depressive symptoms, so this study also evaluated the participants’ depressive symptoms. The PHQ-9 was used to evaluate the participants’ emotional state over the previous two weeks. This questionnaire has nine items that reflect major depressive disorder, based on the DSM-IV standard. Each item is divided into zero to three points, with a total score going from zero to 27 points [13]. The good reliability and validity of the PHQ-9 has been confirmed in the Chinese adult population [22]. In this study, we used the Chinese edition of the PHQ-9 to assess the depressive symptoms of all participants.

#### Summary of assessment instruments

The aforementioned four assessment instruments were integrated into a case report form. Ten assessors—including psychiatrists and graduate students majoring in psychiatry and psychology—were organised to conduct the onsite screening. They were responsible for the informed consent process, collecting patients’ general information and clinical data, and guiding patients as they completed the self-evaluation scale according to standard guidelines. Before screening began, all assessors were trained to ensure the consistency and the quality of the research programme, process, and scales in each hospital. During the screening process, three senior psychiatrists were responsible for monitoring and checking the quality of the case report.

### Statistical analysis

Two independent researchers simultaneously entered data into EpiData3.1. The statistical analysis was conducted using SPSS version 24.0 for Windows. All statistical tests were performed at an alpha level of 0.05. We first explored the data distribution. Continuous variables were examined for normality using skewness and kurtosis within -3 to 3 criteria. Normal data were presented as mean and standard deviation (SD), and non-normal data were presented as median and interquartile range (IQR). Categorical variables were described as frequencies and percentages. The demographic and clinical information of groups of patients with and without SFD were assessed using Student’s t-tests or Mann Whiney U tests for continuous variables and the Chi-square test for categorical variables.

Binary logistic regression analyses were conducted with medical expenses, doctor visit patterns, and whether sick leave days were requested as the dependent variables. The variables with significant differences between groups were selected as independent variables. Because the data for medical expenses have no normal distribution, linear regression analysis is not applicable. Therefore, according to the 50% value (5000 Yuan) of the data, the medical expenses were divided into two groups, namely, the low-cost group (≤ 5000 Yuan) and the high-cost group (> 5000 Yuan), and logistic regression analysis was used. The enter method was used for all regression analyses. Two-tailed estimates of effect and 95% confidence interval were reported for all regression coefficients.

## Results

### Comparison of general information between patients with and without SFD

Among the three hospitals, there were 1600 patients who met the inclusion criteria and agreed to participateto the study. Total 1,497 valid questionnaires were obtained after 103 invalid questionnaires were excluded. The characteristics of all participants are shown in Table 1. The mean age of the participants was 44.40 years (SD = 13.3). Of all subjects, females accounted for 54.84%, while 79.8% of subjects were married, 49.0% were employed, and 29.73% had undergraduate qualifications or above. The distribution of participants from the three hospitals was 32.06%, 33.93% and 34.01%, respectively, with 32% of all participants coming from cardiology, 34.20% from neurology, and 33.80% from gastroenterology.

**Table 1:** Comparison of general information between patients with and without SFD

Items	Total N = 1497	Patients without SFD n = 1242	Patients with SFD n = 255	χ^2^ /t /z	P
Gender				3.30	0.07
Male	626 (45.16%)	574 (46.20%)	102 (40.00%)		
Female	821 (54.84%)	668 (53.80%)	153 (60.00%)		
Age(years)					
M (SD)	44.4 (13.30)	44.55 (13.36)	43.67 (13.10)	0.97	0.33
Marital status				3.27	0.07
Married	1195 (79.8%)	1002 (80.7%)	193 (75.7%)		
Unmarried	302 (20.2%)	240 (19.3%)	62 (24.3%)		
Educational level				3.95	0.41
Junior middle school (9 years)	293 (19.57%)	250 (20.1%)	43 (16.9%)		
Senior High/Technical Secondary School (12 years)	339 (22.65%)	278 (22.4%)	61 (23.9%)		
Junior College (14 years)	267 (17.83%)	359 (28.9%)	86 (33.7%)		
Undergraduate and above (≥ 15 years)	445 (29.73%)	131 (10.5%)	22 (8.6%)		
Employment status				8.80	0.003
Employee	733 (49.0%)	630 (50.8%)	103 (40.6%)		
Un-employee	762 (51.0%)	611 (49.2%)	151 (59.4%)		
Doctor-visiting condition				10.64	< 0.001
First visit	799 (53.4%)	688(55.4%)	111(43.5%)		
Repeat visit	698 (46.6%)	554(44.6%)	144(56.5%)		
Visiting hospital				3.26	0.20
Hospital 1	480 (32.06%)	386 (31.1%)	94 (36.8%)		
Hospital 2	508 (33.93%)	427 (34.4%)	81 (31.8%)		
Hospital 3	509 (34.01%)	429 (34.5%)	80 (31.4%)		
Visiting department				3.66	0.16
Cardiology	479 (32.00%)	401 (32.3%)	78 (30.6%)		
Neurology	512 (34.20%)	434 (34.9%)	78 (30.6%)		
Gastroenterology	506 (33.80)	407 (32.8%)	99 (38.8%)		
Duration of symptom(Month)	3 (1, 24)	3 (1, 24)	6 (1, 35)	2.17^a^	0.03
Median (IQR)					

^a^ Mann- Whitney U test for non-normal distribution data

*SFD* somatoform disorder, *M* mean, *SD* standard deviation, *IQR* interquartile range

Bold values indicate that the p-value is statistically significant

According to the SQSS cut-off, subjects with scores equal to or greater than 29 were considered as having a SFD diagnosis. Among the 1,497 subjects, 255 subjects were recognised as being SFD. The overall detection rate of patients with SFD was 17.03%. The detection rate of patients with SFD among the three hospitals was 19.58%, 15.94% and 15.72%, respectively, and the detection rate was 16.28% in patients from cardiology, 15.23% in patients from neurology, and 19.57% in patients from gastroenterology.

As shown in Table 1, there were significant differences in employment status, doctor visits, and duration of symptoms between patients with and without SFD (all *p* < 0.05). No significant differences were found in age, gender, educational level, hospital visits, and hospital department visits between the two groups (all *p* > 0.05).

### Comparison of clinical characteristics and economic burden between patients with and without SFD

As shown in Table 2, the patients with SFD showed higher number of days of sick leave, medical expense, PHQ-15 total score, PHQ-9 total score, and SQSS total score than patients without SFD (all *p* < 0.05). In addition to the SQSS total score, the patients with SFD also showed higher scores in the four-dimensional scores on the SQSS (somatic symptoms, negative perceptions, illness behaviour, and social functioning) than patients without SFD (all *p* < 0.05).

**Table 2:** Comparison of clinical characteristics and economic burden between patients with and without SFD

Items	Total N = 1497	Patients without SFD n = 1242	Patients with SFD n = 255	χ^2^ /t /z	P
PHQ-9 total score M (SD)	4.90 (4.63)	3.86 (3.68)	10.10 (5.19)	22.91	< 0.001
PHQ-15 total score M (SD)	7.87 (5.16)	6.55 (4.10)	14.29 (4.98)	26.38	< 0.001
SQSS total score M (SD)	17.55(12.69)	12.98 (7.30)	39.81 (9.38)	5.073	< 0.001
Somatic symptom M (SD)	8.32 (5.59)	6.63 (4.10)	16.5 (4.54)	34.28	< 0.001
Negative perceptions Median (IQR)	2 (0, 4)	1 (0, 3)	7 (4, 10)	20.00^a^	< 0.001
Illness behaviour M (SD)	3.95 (3.86)	2.81 (2.74)	9.62 (3.62)	33.65	< 0.001
Social function Median (IQR)	1 (0, 4)	1 (0, 3)	6 (4, 8)	20.25^a^	< 0.001
Medical expense(Yuan/Month)	5000 (1250, 20000)	5000 (1200, 20000)	10000 (2500, 27750)	8.39^a^	0.03
Median (IQR)					
Number of sick leave days Median (IQR)	2 (1, 8)	1(1, 7)	3 (1, 21)	1.07^a^	0.05

^a^ Mann- Whitney U test for non-normal distribution data

*SFD* somatoform disorder, *PHQ-9* Patient Health Questionnaire-9, *PHQ-15* Health Questionnaire-15, *SQSS* self-screening questionnaire for somatic symptoms, *M* mean, *SD* standard deviation, *IQR* interquartile range

Bold values indicate that the p-value is statistically significant

**Fig. 1:**
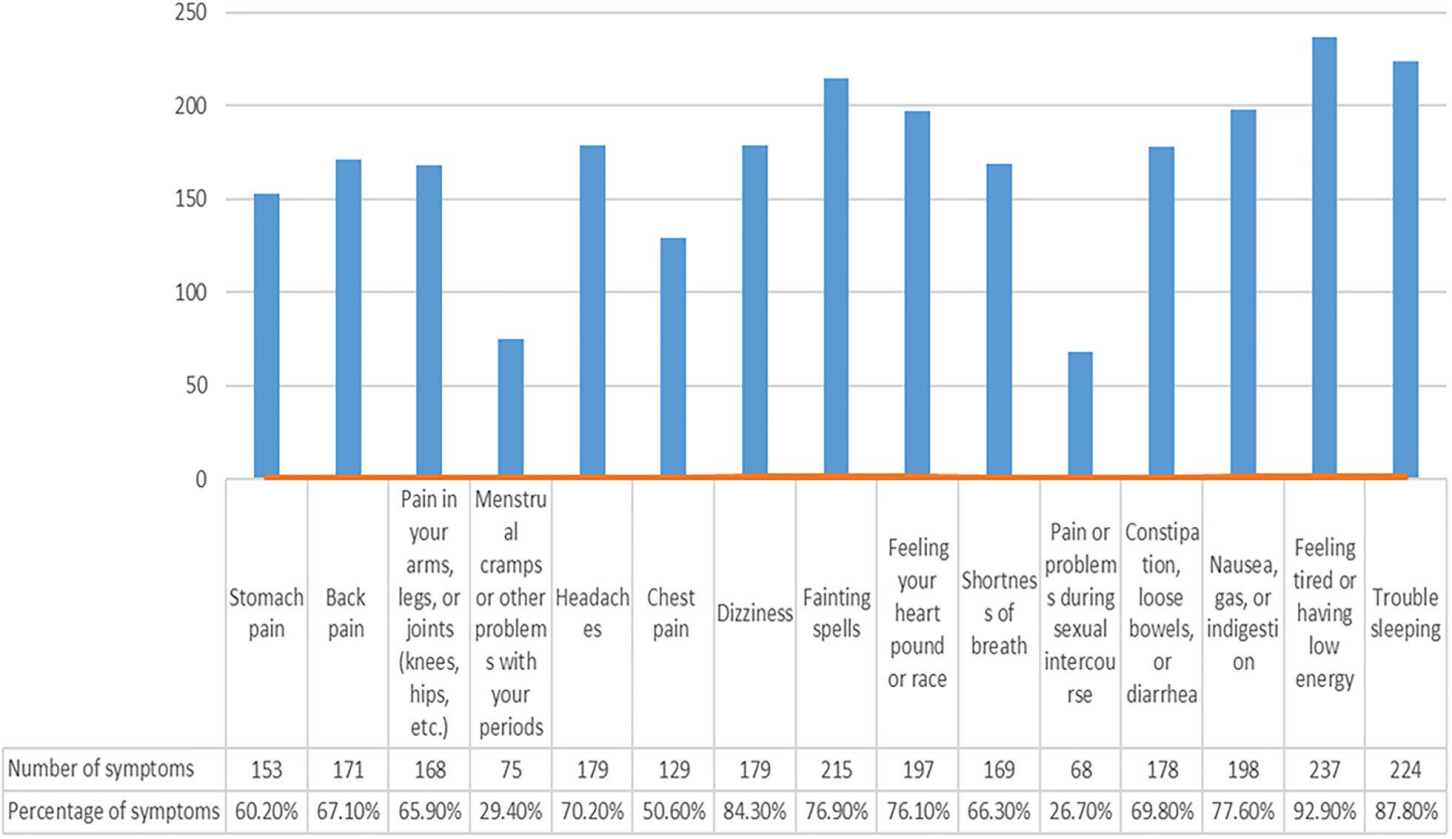
The distribution of somatic symptoms of PHQ-15 in patients with SFD

Among the four dimensions of the SQSS, the somatic symptoms dimension scored the highest (16.5 ± 4.54). Among all subjects, 32.67% (489/1497) patients had multiple somatic symptoms based on the cutoff value of PHQ-15 (≥ 10). We then further scored the somatic symptoms on the PHQ-15 for subjects with SFD to display the distribution of the physical symptoms. As shown in Fig. 1, the three most common symptoms among these subjects were ‘feeling tired or having low energy’ (92.9%), ‘trouble sleeping’ (87.8%) and ‘dizziness’ (84.3%). The subjects from different departments showed alteration on the three most common symptoms. The three most common symptoms among the SFD patients from cardiology department were ‘feeling tired or having low energy’ (90.5%), ‘trouble sleeping’ (85.3%) and ‘feeling your heart pound or race’ (80.5%); among those from neurology department were ‘dizziness’ (91.2%), ‘feeling tired or having low energy’ (84.5%) and ‘trouble sleeping’ (82.8%); among those from gastroenterology department were ‘feeling tired or having low energy’ (91.5%), ‘stomach pain’ (90.3%), and ‘nausea, gas, or indigestion’ (85.3%).

### Logistic regression analysis of medical expense, multiple doctor visits, and requests for sick leave in patients with SFD

Results showed that patients with SFD had higher medical expense, more doctor visits, and more requests for sick leave days. Then, we further explored the factors potentially related to these adverse consequences by using binary logistic regression analyses. Based on the comparisons between the above two groups, we included gender, marital status, duration of symptoms, employment status, and scores measuring the four dimensions of the SQSS as predictor variables in the regression analysis.

The results of ANOVA tests showed the regression model for medical expenses was significant (F = 64.25, *p* < 0.001). As shown in Table 3, the duration of symptoms and score for social functioning were positively associated with higher medical expenses, with OR = 1.009 (95% CI 1.005, 1.013) and OR = 1.082 (95% CI 1.012, 1.158), respectively. Compared to males, females had a reduced risk of higher medical expenses (OR: 0.719, 95% CI 0.514, 1.005). Unmarried individuals also had a reduced risk of higher medical expenses than married individuals (OR: 0.408, 95% CI 0.256, 0.651).

**Table 3:** Binary Logistic regression of higher medical expense, repeat doctor- visiting, and asking for sick leave

Variables	Higher medical expense (> 5000 yuan/month)		Repeat doctor-visiting		Asking for sick leave	
	OR (95% CI)	p	OR (95% CI)	P	OR (95% CI)	P
Male	1		1		1	
Female	0.719 (0.514,1.005)	0.05	0.882 (0.696, 1.118)	0.30	0.743 (0.578, 0.954)	0.02
Married	1		1		1	
Unmarried	0.408 (0.256, 0.651)	< 0.01	0.780 (0.583, 1.044)	0.10	2.339 (1.744, 3.136)	< 0.01
Employee	1		1		1	
Un-employee	1.353 (0.969,1.889)	0.08	1.468 (1.162, 1.855)	< 0.01	0.881 (.473, 1.640)	0.69
Duration of symptom	1.009 (1.005,1.013)	< 0.01	1.009 (1.006, 1.012)	< 0.01	0.997 (0.994, 1.000)	0.06
Somatic symptom	1.004 (0.964,1.045)	0.86	1.004 (0.977, 1.032)	0.79	0.982 (.953, 1.011)	0.22
Negative perceptions	1.014 (0.947,1.086)	0.69	0.966 (.920, 1.013)	0.15	0.982 (0.934, 1.033)	0.49
Social function	1.082 (1.012,1.158)	0.02	1.010 (0.963, 1.058)	0.96	1.055 (1.005, 1.108)	0.03
Illness behaviour	0.999 (0.944,1.058)	0.99	1.065 (1.022, 1.110)	< 0.01	1.060 (1.015, 1.107)	< 0.01

*OR* odds ratio, *CI* confidence interval

Bold values indicate that the p-value is statistically significant

The regression model for doctor visits was significant (F = 74.78, *p* < 0.001). Compared to employees, non-employee status was related to multiple doctor visits (OR: 1.468, 95% CI 1.162, 1.855). Duration of symptoms and the score for illness behaviour were also positively associated with multiple doctor visits, with OR = 1.009 (95% CI 1.006, 1.012), respectively (Table 3).

When requests for sick leave days was set as the dependent variable, the regression model was significant (F = 74.78, *p* < 0.001). As shown in Table 3, the scores measuring social functioning and illness behaviour in the SQSS were positively associated with requests for sick leave days, with OR = 1.055 (95% CI 1.005, 1.108) and OR = 1.060 (95% CI 1.015, 1.107), respectively.

## Discussion

This study identified patients with SFD in three general hospitals using the SQSS scale, a newly developed self-screening instrument for SFD. The results indicated that the detection rate was 17.03%, suggesting that SFD is prevalent among patients in general hospitals in China. As we expected, patients with SFD had higher medical expenses, utilised more health care (made repeated visits to doctors), and requested more sick leave days than to those without SFD. Furthermore, we found several factors associated with these negative consequences. These findings suggest that doctors in general hospitals can identify SFD patients in a timely manner based on SQSS scores and specific characteristics of SFD, and provide appropriate interventions.

### Detection rate of individuals with SFD in the general hospital

The prevalence of SFD was much higher in the primary care patient population than in the general population, especially in some certain patient. Populations with functional disorders, including fibromyalgia, irritable bowel syndrome, and chronic fatigue syndrome [23, 24]. The estimated prevalence of SFD based on the DSM-IV criteria was found to be 16.1% in a Dutch population of those consulting general practitioners [3]. One recent review reported that the prevalence of SFD and MUS in primary care is greater than 20% in Western countries [4]. Current study found a prevalence of SFD (estimated throughout SQSS) of 17.05% in the three general hospitals, which was similar to that found in the above-mentioned studies. The SQSS scale used in this study has four dimensions, and is more specific for SFD than the PHQ-15. This last scale offer to consider a 58.5% specificity, and an inflated SFD prevalence of 28% [21]. In this study, if we use PHQ-15 to screen SFD, the detection rate was as high as 32.67%, which also shows that PHQ-15 is not as specific as SQSS. We found no differences in the prevalence of SFD among the three hospitals and the three different departments that we studied. These results suggest that SFD is a common mental problem in Chinese general hospitals, as is the case in Western countries. Thus, this patient population in the general hospitals should be paid more attention in order to reduce medical cost.

### Demographic and clinical characteristics of patients with SFD

Previous study showed that female is more likely to develop SFD than male in general population [23]. However, we did not find the significant gender difference in both groups with and without SFD. This inconsistent finding might be due to the different sample populations. This study found unemployed subjects account for a higher percentage in patients with SFD. Previous studies have found that the prevalence of SFD and MUS among subjects without work and retirees was much higher than in those who were employed [5, 19, 25, 26]. These findings indicate that ageing, a low income, and low social status are associated with patients’ physical and psychological health.

It has been reported that the patients with SFD and related illnesses often utilise more health care due to their persistent health complaints, and that they make higher numbers of visits to doctors, with a mean of 28.02 doctor visits each year [8]. In this study, we found that repeat visits accounted for majority of all medical consultations in patients with SFD, and that this group reports a longer duration of somatic symptoms than those without SFD. On the one hand, this phenomenon is consistent with the clinical characteristics of chronic course and fluctuation of symptoms in SFD. Due to the painful somatic symptoms, chronic illness course, and unclear cause of SFD, patients have to visit doctors repeatedly to seek assurance [27]. On the other hand, this behaviour might be explained by the cognitive-behavioural model (CBT) of MUS/SFD, which assume that SFD patients have distorted perception on somatic symptoms. Because of the dysfunctional beliefs, they often deny doctors’ explanations, diagnosis, and referrals [28]. 

The SQSS scale includes four dimensions: somatic symptoms, negative perceptions, illness behaviour, and social function. In addition to severe somatic symptoms, patients with SFD also present more dysfunctional perceptions and behaviours regarding illness, as well as decreased social functioning, compared to those without SFD. This finding is consistent with the diagnostic criteria for SSD in DSM-5 [16]. A key change from SFD in DSM-IV to SSD in DSM-5 is the introduction of psychological symptoms (impaired thoughts, emotions, and behaviour) [29]. Thus, the current results suggest that the SQSS may be a useful tool to focus on multidimensionality of the assessment, and to be consistent with DSM-5 for the early detection of SSD and related disorders.

In this study, the patients with SFD also showed higher scores on the PHQ-15. When we analysed the reliability and validity of the SQSS scale, we also found that the total SQSS score was positively correlated with the total PHQ-15 score [20]. We examined the somatic symptoms distribution in patients with SFD using PHQ-15, and found three most common physical symptoms: ‘feeling tired or having low energy’, ‘trouble sleeping’ and ‘dizziness’. This finding is consistent with previous studies showing that nonspecific somatic symptoms are common manifestations of SFD and should be noticed by general practitioners [21].

Depressive and somatic symptoms often occur simultaneously and interact with each other [30, 31]. One study found that 17% of patients with SFD had a depressive disorder, and 54% of patients with depressive disorder had SFD [3]. In this study, we also found that patients with a higher PHQ-9 score had a higher prevalence of SFD than patients with a lower PHQ-9 score. From the perspective of CBT, distress intolerance is as a key theme of MUS/SFD maintenance. Improving negative emotional states such as sadness and anger in the treatment of SFD is effective for the amelioration of patients' somatic symptoms [28]. One study explored the involvement of negative emotional intensity in stressful social situations in relation to the effects of CBT for patients with persistent somatoform pain. The results showed that CBT can improve negative emotional intensity during stressful social situations in these patients. In addition, there is a moderate positive correlation between changes in negative emotional intensity before/after treatment and clinical symptoms of pain. This finding suggests that the management of negative emotions such as depression and anxiety in SFD patients should be addressed as a priority treatment prior to the treatment of SFD. In summary, the PHQ-15, PHQ-9 and SQSS can be used simultaneously to make up for the deficiencies of a single scale, which will improve accuracy in the identification of SFD in clinical practice and to inform treatment decisions. It is noted that previous studies have shown that SFD often combined with anxiety [33]. We could not assess the anxiety symptom in this study. In the future study, the anxiety symptom for the SFD patients should be assessed.

### Factors associated with medical expenses, visits to doctors, and sick leave in patients with SFD

In this study, participants with SFD had greater medical expenses, visiting doctors multiple times, and requested more sick leave days compared to those not at risk of SFD. Typically, patients with SFD and MUS had high medical costs and made frequent visits to doctors, due to their long duration of illness and complex medical experiences. In addition to these direct costs, they also undergone a heavy indirect cost burden, largely as a result of lost productivity (increased sick leave) [34]. These negative consequences may further affect the quality of life in this patient group. To better manage SFD in clinics, we further identified factors associated with these conditions.

We found that duration of symptoms and the SQSS social functioning score were associated with higher medical expenses. SFD patients typically exhibit a chronic course of disease. It is estimated that 20% to 25% of patients with acute somatic symptoms will develop into chronic somatic diseases [35]. Moreover, these people are more sensitive to physical symptoms and insufficient social support, so they are more inclined to seek medical care, with associated high medical expenses [36]. Impairment of social functioning is an essential criterion for a diagnosis of SFD [2]. The SQSS measured individuals’ social functioning, including their ability to work and maintain their home duties. Our results showed that poorer social functioning was associated with higher medical costs. This is consistent with a previous study conducted in Germany, which found that symptom-related disability may be a potential influencing factor in increasing healthcare use [8]. Moreover, the behaviour of repeatedly seeking medical care not only increases medical costs, but also contributes to the development and maintenance of SFD, because it may delay the adequate coping strategies’s development [37]. Although previous studies have shown that female and unmarried people are at higher risk of SFD and related diseases [5, 10, 21], this study found that their risk of incurring high medical expenses is lower than that of male and married individuals, respectively. This may be due to the lower income and social support of female and unmarried individuals, which limits their ability to seek medical care. The Chinese Third National Health Service survey showed that female patients’ medical expenditure was less than male. This finding indicated that gender difference in medical costs may be related to lower social position and less sufficient economic security of female population [38].

Visiting doctors in the outpatient clinics of general hospitals is the main way of seeking medical care in China. We found that unemployment, duration of symptoms and illness behaviour were associated with visiting doctors more than twice. Obviously, a longer duration of symptoms was related to multiple doctor visits. Previous studies using the PHQ-15 as screening tool have found that the unemployed and retired populations have more somatic symptoms than those who are in work; people which are unemployed may pay more attention to their somatic symptoms and have more time to seek medical care [26]. Illness behaviour, as measured by seeking health care and number of medical consultations, is a significant and persistent manifestation of SFD. A previous study has highlighted that increased outpatient physician visits were mainly driven by illness behaviour in patients with MUS [9]. It is possible that these patients’ persistent somatic symptoms are inevitably related to repeated visits to doctors, particularly when no cause is identified for the patient’s symptoms.

Sick leave is an important component of the indirect costs to patients. It has been reported that patients with severe SFD exhibited a significantly higher number of days of sick leave than patients with mild or moderate level of symptoms [8]. In this study, we found female gender, being unmarried, longer duration of symptoms, illness behaviour and poorer social functioning were associated with more sick leave days. It is possible that females and unmarried people took more sick leave days due to a lack of social support [36, 39]. The duration of symptoms may be related to both direct and indirect costs in patients diagnosed with SFD. Illness behaviour and poor social functioning may be the main reason for these patients to seek medical care and ask for sick leave days. In conclusion, social functioning and illness behaviour, as measured by the SQSS, may be associated with higher medical expenses, greater numbers of visits to doctors, and more sick leave. Thus, a timely psychological evaluation is imperative in this population.

### Limitations

This study has several limitations. First, given our limitations in terms of human and material resources, we screened patients in three departments of three general hospitals using convenience sampling, which may have led to selection bias. Second, the screening instruments used in this study were self-reported, and there may have been an information bias when patients responded to some questions. The noisy environment in the outpatient lobby may have affected patients’ thought processes, thereby leading to deviation in their results. Third, due to its cross-sectional design, this study cannot explain whether there was a causal relationship between the diagnosis of SFD and its related factors. Thus, the current findings need to be interpreted with caution. Last, we built four dimensions of SQSS based on the diagnostic criteria of DSM-5 for SSD, but used structured clinical interview for DSM-IV-TR AXIS I disorder [SCID-I/P] as a diagnostic tool to measure the reliability and validity of SQSS [20]. Although SFD and SSD are highly coincident in clinical manifestations, the effect of SQSS in screening SSD cannot be determined. In order to be consistent with the diagnostic system of DSM-5, we need to further verify the reliability and validity of SQSS screening SSD in the future studies using a large and randomly selected patient population in the outpatient clinics.

## Conclusions

In conclusion, this study's findings indicate that those with SFD account for 17.03% of all patients in general hospitals. General nonspecific somatic symptoms are also frequently present in patients with SFD. In addition to somatic symptoms, these patients present with dysfunctional perceptions and behaviour, as well as impaired social function. Depressive symptoms are also common in patients with SFD. Further, these patients have high medical expenses, make multiple visits to doctors and many requests for sick leave days. This study has identified specific characteristics among patients with SFD, which will help to improve the recognition of SFD as early as possible in general hospitals, and ultimately improve the diagnosis rate and reduce the economic burden associated with this condition.

### Supplementary Information

**Additional file 1: Table S1**. The content of self-screening questionnaire for somatic symptoms (SQSS).

## Data Availability

All data generated or analysed during this study are included in this published article.

## Abbreviations

SFD: Somatoform disorders
MUS: Medically unexplained symptoms
SSD: Somatic symptom disorder
SSS-CN: Somatic Symptom Scale-China
SQSS: Self-screening questionnaire for somatic symptoms
PHQ-15: Patient health questionnaire-15
PHQ-9: Patient health questionnaire-9
IQR: Median and interquartile range
M: Mean
SD: Standard deviation
OR: Odds ratio
CI: Confidence interval
CBT: Cognitive-behavioural therapy
