# Supplementary material for: Characteristics and economic burden of patients with somatoform disorders in Chinese general hospitals: a multicenter cross-sectional study
Source: Ann Gen Psychiatry. 2023 Aug 12;22:30. doi: 10.1186/s12991-023-00457-y (PMC10423408; doi:10.1186/s12991-023-00457-y)
Supplement: Supplementary file 1 — Additional file 1: Table S1. The content of self-screening questionnaire for somatic symptoms (SQSS). [file 12991_2023_457_MOESM1_ESM.docx]

**Additional file 1: Table S1. The content of self-screening questionnaire for somatic symptoms (SQSS)**

| Item | Content | Degree | | | | |
| --- | --- | --- | --- | --- | --- | --- |
|  |  | Never  0 | Occasion  1 | Sometimes  2 | Usually  3 | Always  4 |
| 1 | I feel pain and discomfort in some part of my body for no reason. |  |  |  |  |  |
| 2 | The symptoms of physical discomfort make me incompetent for work. |  |  |  |  |  |
| 3 | The idea of death makes me nervous and frightened. |  |  |  |  |  |
| 4 | I can't take care of myself because I'm not feeling well. |  |  |  |  |  |
| 5 | I have sleep problems for unknown reasons. |  |  |  |  |  |
| 6 | I check my body over and over again to find possible problems as soon as possible. |  |  |  |  |  |
| 7 | The current physical discomfort has reduced my ability to do housework. |  |  |  |  |  |
| 8 | All kinds of physical discomfort have reduced my quality of life. |  |  |  |  |  |
| 9 | Because I am worried about my health, I continue to collect relevant information through various channels, such as the Internet. |  |  |  |  |  |
| 10 | I feel panic, palpitation or chest tightness for no reason. |  |  |  |  |  |
| 11 | I have constipation, loose stool or diarrhea for unknown reasons. |  |  |  |  |  |
| 12 | I feel tired, painful and uncomfortable. |  |  |  |  |  |
| 13 | I get nervous when I hear that someone has a disease. |  |  |  |  |  |
| 14 | I feel tired or listless for unknown reason. |  |  |  |  |  |
| 15 | I frequently go to the hospital to see a doctor or do various examinations to identify my illness. |  |  |  |  |  |
| 16 | Concerned about my health, I constantly seek assurance or comfort from my family or friends. |  |  |  |  |  |
| 17 | If I don't feel well, it means I have a serious physical disease |  |  |  |  |  |
| 18 | If someone else is not feeling well, then I will feel uncomfortable. |  |  |  |  |  |
| 19 | I've seen many doctors in different departments, or other therapists. |  |  |  |  |  |
| 20 | I inexplicably appear nausea, heartburn, flatulence and other indigestion symptoms. |  |  |  |  |  |
| 21 | News related to death (such as funerals or obituaries) makes me nervous and frightened. |  |  |  |  |  |
| 22 | I feel dizzy for no reason, or even faint. |  |  |  |  |  |
| Total score | | | | | |  |

Dimensions: Somatic symptoms include item 1, 5,10, 11, 12, 14, 20, 22; Dysfunctional belief includes item 3, 13, 17, 18, 21;

Illness behaviour includes item 6, 9, 15, 16, 19; Social function includes item 2, 4, 7, 8
